# Identification of drug-resistant *Salmonella* from food handlers at the University of Gondar, Ethiopia

**DOI:** 10.1186/1756-0500-7-545

**Published:** 2014-08-18

**Authors:** Legesse Garedew-Kifelew, Nishanwork Wondafrash, Amsalu Feleke

**Affiliations:** Faculty of Veterinary Medicine, University of Gondar, Gondar, Ethiopia; Institute of Public Health, University of Gondar, Gondar, Ethiopia

**Keywords:** Antimicrobial susceptibility, Food handlers, Prevalence, *Salmonella*, Ethiopia

## Abstract

**Background:**

*Salmonella* species are among the most common food borne pathogens worldwide and their infection is one of the major global public health problems. During the last decade, multidrug-resistant S*almonella* species have increased to a great deal, especially in developing countries. The prevalence and antimicrobial susceptibility pattern of *Salmonella* isolates among food handlers at the University of Gondar, Ethiopia, were described in the current investigation.

**Method:**

A cross-sectional study was conducted from February to June, 2013 at the University of Gondar. Stool samples from selected volunteer food handlers were collected and analyzed complemented with questionnaire. Standard isolation, identification and biochemical tests were performed to identify *Salmonella* isolates. Antimicrobial susceptibility tests were also carried out on each isolate using Kirby-Bauer disc diffusion method. The data was entered into Epi info version 3.5.4 and analyzed using SPSS version 21.

**Result:**

Out of 423 food handlers participated, 303(71.6%) were females. Almost two-third (71.4%) of food handlers had no previous medical checkup to *Salmonella* infection and only 24(5.7%) of them were certified as food handlers. Thirteen (3.1%) food handlers were found to be positive for *Salmonella* isolates. The results of antimicrobial susceptibility test in the current research revealed that from a total of 13 isolates; 9(69.2%), 8(61.5%), 6(46.2%) and 6(46.2%) of the isolates were resistant to amoxicillin, ampicillin, nitrofurantoin and tetracycline, respectively. In addition, nearly half (46.2%) of the isolates were multidrug-resistant. However; all of them were sensitive for both ceftriaxone and gentamycin.

**Conclusion:**

This study indicated that drug resistant including multidrug-resistant *Salmonella* isolates were circulating among food handlers at the University of Gondar. These *Salmonella* positive food handlers pose significant risk of infection to the university community particularly to the student population. It is essential to implement food handlers training on food safety, conduct periodic medical screening and continuous monitoring of food handlers at the study university.

## Background

Infection due to *Salmonella* is a public health problem both in developed and in developing countries. WHO estimated that, globally 3 million *Salmonella* associated deaths have been annually reported. *Salmonella* infection most commonly occurs in countries with poor standards of hygiene in food preparation, handling and sewage disposal [1].

Food borne salmonellosis often follows consumption of contaminated animal products, fruits and vegetables as well as contamination through unclean work surfaces used to prepare foods. Food can also be contaminated by food handlers who do not thoroughly wash their hands with soap after handling raw foods or after using the bathroom. The carrier states of humans are of concern to the food manufacturing and food service institutions because of the risk of contamination of food [2].

The widespread nature of salmonellosis and the spread of antibiotic resistance are of major concern for both developed and developing countries. An example of the global threat of antibiotic resistance is the multidrug-resistant (MDR) *Salmonella,* resistant for three or more antimicrobial classes. MDR isolates have been widely reported in Europe and America from travelers and adopted children [3]. MDR in both the hospital and community environment are important concern to the clinician, patients and the pharmaceutical industries. Antimicrobial drugs misuse, drugs prescription without susceptibility test, self-medication and long duration of hospitalization was suggested to augment the problem of MDR in developing nations [4].

The true incidence of salmonellosis in humans is difficult to evaluate because of lack of an epidemiological surveillance system in different areas, particularly in developing countries. But a few studies indicated the widespread occurrence and distribution of *Salmonella* in Ethiopia. According to reports [5–7], in recent years the number of out breaks of *Salmonella* in humans and its antimicrobial resistance has increased considerably in the country. Despite the challenge, research conducted about food salmonellosis among food handlers and its antimicrobial susceptibility pattern is scarce in Ethiopia particularly in Gondar. Therefore; this research was initiated to determine the prevalence and antimicrobial susceptibility pattern of *Salmonella* isolates among food handlers working at the University of Gondar students’ dining rooms to recommend appropriate prevention and control measures.

## Methods

### Study area

The study was carried out on food handlers (those participated in food preparation, dispatch, store and related services) of University of Gondar students’ dining rooms in all campuses. Laboratory investigation was done in the Veterinary Public Health and Microbiology Laboratory, University of Gondar. The University has a latitude and longitude of 12°36’N37°28’E with an elevation of 2133 meters above sea level and located 730 km north of Addis Ababa [8]. University of Gondar currently enrolled more than 26,000 students out of which more than half of them are getting dining services in the dining rooms included in this study. A total of 664 food handlers are working in these students dining rooms.

### Sample size determination and sampling procedure

The sample size was determined by using a single population proportion formula [9] considering the following assumptions: Zα/2 = 1.96 for the standard scale of 95% level of confidence, level of precision = 5%, P = 0.5


The total sample size was 423 with 10% non response rate included.

### Sampling and data collection procedure

Simple random sampling using lottery method was used to select the study subjects. Complete list of food handlers was obtained from human resource management of University of Gondar. Socio-demographic data was gathered by using structured pretested questionnaire and face to face interview. Stool specimens were collected from food handlers with a suitable labeled wide-mouthed plastic container and clean wooden applicator stick. Specimens were immediately transported to laboratory using ice box.

### Bacteriological examination

Bacteriological examination was carried out based on standard procedures previously described [10]. Briefly: twenty five grams of stool sample was homogenized in 225 ml of buffered peptone water (BPW, Oxoid, England) within a sterile stomacher bag. The homogenate was mixed well using a laboratory blender (Stomacher 400, Seward, England) for 1 minute and incubated for 24 hours at 37°C. Then, 1 ml, 1 ml and 0.1 ml aliquot of the enrichment broths was transferred aseptically into 10 ml of Selenite Cystine (SC), 10 ml of Tetrathionate (T) and 10 ml of Rappaport-Vassilliadis (RV) broth, and incubated for 24 hours at 37°C, 37°C and 42°C, respectively. Following incubation, a loopful of each culture was streaked onto Brilliant Green Agar (BGA, Oxoid, England) and Xylose Lysine Deoxycholate (XLD, Oxoid, England) agar plates and incubated at 37°C for 24 to 48 hours. The plates (BGA and XLD) were examined for the presence of characteristics *Salmonella* colonies. A single positive colony showing red color with a black center on XLD and red color on BGA agars were subjected for biochemical tests for confirmation.

### Biochemical tests

Identification of *Salmonella* was performed by subjecting presumptive colonies onto Triple Sugar Iron (TSI) agar, Lysine Iron agar, Methyl Red (MR) broth, Voges-Proskauer (VP) broth, Urea broth, Indole test, and Citrate utilization tests and incubated for 24 to 48 hours at 37°C. Colonies producing an alkaline slant with acid butt and hydrogen sulfide production on TSI, positive for lysine, negative for urea hydrolysis, negative for Indole test, negative for VP, positive for citrate utilization and positive for MR test were considered to be *Salmonella* [10]. Finally, all of the confirmed *Salmonella* isolates were examined for antimicrobial susceptibility.

### Antimicrobial susceptibility test

All isolates were tested by Kirby-Bauer disk diffusion method using guidelines established by the Clinical and Laboratory Standards Institute (CLSI) [11]. In brief, by taking pure isolated colonies, bacterial suspension in test tubes was adjusted and compared to 0.5McFarland turbidity standards. The diluted bacterial suspension was then transferred to Mueller-Hinton agar plate using a sterile cotton swab and the plate was seeded uniformly by rubbing the swab against the entire agar surface followed by 24 hours incubation. Antibiotic impregnated discs were then applied to the surface of the inoculated plates using sterile forceps. The plates were then incubated aerobically at 37°C for 24 hours. *E. coli* (ATCC 25922), which was susceptible to all tested drugs, was used for quality control. A total of 9 selected antibiotic disks (Oxoid, UK) including amoxicillin (AML) 2 μg, ampicillin (AMP) 10 μg, cephalothin (KF) 30 μg, ceftriaxone (CRO) 30 μg, gentamicin (CN) 10 μg, nalidixic Acid (NA) 30 μg, sulphamethoxazole-trimetoprime (SXT) 25 μg, teteracycline (TE) 30 μg and nitrofurantoin (F) 100 μg were applied. Finally, the zone of inhibition was measured including the disk diameter and the susceptible, intermediate and resistant categories were assigned on the basis of the critical points recommended by the CLSI.

### Study variables

Prevalence of *Salmonella* and their antimicrobial susceptibility patterns were dependent variables whereas sex, age, educational background, service year, hand washing habit, periodical medical examination, and food hygiene training status were independent variables considered in the current study.

### Data processing and analysis

Data were cleaned and entered using Epi-Info version 3.5.4. The data then was transferred and analyzed using SPSS version 21. Descriptive statistics such as percentage were applied to compute the data.

### Ethical consideration

Ethical clearance was obtained from Institutional Review Board of Institute of Public Health, College of Medicine and Health Sciences, University of Gondar. Written informed consent was obtained from all study participants.

## Results

### Socio-demographic characteristics of the study participants

Overall, 423 food handlers were participated in this study with mean age and standard deviation of 33.5 ± 10.5. Three hundred three (71.6%) of the participants were females. Of the total participants, 24(5.7%), 248(58.6%) and 151(35.7%) of them were trained and certified, trained but not certified and received no training for food preparation and handling, respectively. Regarding regular periodical medical examination for infectious diseases, only 121(28.6%) was confirmed taking periodical medical check-up. Evaluation of their service year in dining rooms indicated that 326(77.1%) food handlers had 2–9 years of services.

### *Salmonella*infection status of food handlers

Stool specimens examination revealed that 13(3.1%) out of 423 participants were infected with *Salmonella*. All of the infected food handlers were females. The highest proportion of infection (4.2%) across age was within the age group of 28–37 years. Of the total *Salmonella* positives, 10(76.9%) were from formally educated group and the highest level of infection (53.8%) was associated with 1–4 grade level as tabulated in Table 1.

**Table 1:** **Prevalence of** ***Salmonella*** **among food handlers at the University of Gondar**

Variables		Total tested	***Salmonella***positive
		Number (%)	Number (%)
Age-group	18-27 years	134(31.7%)	4(3%)
	28-37 years	143(33.8%)	6(4.2%)
	38-47 yeas	101(23.9%)	0%
	48-57 years	38(8.9%)	2(5.3%)
	>58 years	7(1.7%)	1(14.3%)
Sex	Male	303	13(4.3%)
	Female	120	0(0.0%)
Educational background	Cannot read and write	11(2.6%)	1(9.1%)
	Read and write only	36(8.5%)	2(5.6%)
	1-4 grades	163(38.5%)	7(4.3%)
	5-8 grades	113(26.7%)	0(0%)
	9-12 grades	95(22.5%)	3(3.2%)
	>12 grades	5(1.2%)	0(0%)
Periodical medical check-up	Yes	121	2(1.6%)
	No	302	11(3.6%)
Service year in dining rooms	*≤* 1 year	45	3(6.7%)
	2-9 years	326	8(2.4%)
	10^+^ years	52	2(3.9%)
Hand washing habit after using a toilet by water only	Yes	318	7(2.2%)
	No	105	6(5.7%)
Hand washing habit before preparing food	Yes	301	5(1.7%)
	No	122	8(6.6%)
Hand washing habit after touching dirty materials	Yes	82	3(3.7%)
	No	341	10(2.9%)
Hand washing habit after using a toilet by soap and water	Yes	272	4(1.5%)
	No	151	9(7.4%)

The isolation rate of *Salmonella* was higher among food handlers who served in the students’ dining rooms for a period of ≤1 years (6.7%) since most of them were not trained in food handling and preparation formally. From a total of 302 food handlers who never undergone periodical medical check-up for the common infectious diseases, 11(3.6%) were *Salmonella* positives. Relatively higher *Salmonella* prevalence was detected in those who did not undertake periodical medical check-up. In addition, those who did not have hand washing habit after using toilet, after touching dirty materials and before starting preparation of food were risk groups from whom relatively higher prevalence of *Salmonella* was recorded.

### Antimicrobial susceptibility patterns of *Salmonella*isolates

Of the 13 *Salmonella* isolates subjected to antimicrobial susceptibility test using a panel of 9 different antimicrobials, 6(46.2%) were multidrug-resistant. On the other hand, all of the isolates were susceptible for both ceftriaxone and gentamycin. Majority of the isolates 11(84.6%) were also sensitive to nalidixic acid and sulphamethoxazole-trimetoprime. But none of the *Salmonella* isolates identified was sensitive to all antimicrobial agents tested. This indicated that all of the isolates were resistant at least to one antimicrobial agent. The highest resistance was exhibited by 9(69.2%), 7(54.8%), 6(46.2%) and 6(46.2%) of the isolates for amoxacillin, ampicillin, tetracycline and nitrofurantoin, respectively, as shown in Tables 2 and 3.

**Table 2:** **Antimicrobial susceptibility patterns of** ***Salmonella*** **isolates from food handlers**

Antimicrobials (μg)	Antibiotic susceptibility patterns		
	Sensitive N(%)	Intermediate N(%)	Resistant N(%)
Amoxacillin (AML_2_)	3(23.1%)	1(7.7%)	9(69.2%)
Ampicillin (AMP_10_)	4(30.8%)	2(15.4%)	7(54.8%)
Cephalothin (KF_30_)	5(38.5%)	3(23%)	5(38.5%)
Ceftriaxone (CRO_30_)	13(100%)	0(0%)	0(0%)
Gentamicin (CN_10_)	13(100%)	0(0%)	0(0%)
Nalidixic acid (NA_30_)	11(84.6%)	0(0%)	2(15.4%)
Sulphamethoxazole-trimetoprim (SXT_25_)	11(84.6%)	0(0%)	2(15.4%)
Tetracycline (TE_30_)	7(53.8%)	0(0%)	6(46.2%)
Nitrofurantoin (F_100)_	7(53.8%)	0(0%)	6(46.2%)

**Table 3:** **Drug resistance profile of** ***Salmonella*** **isolates from food handlers**

Resistance profile	No of isolates with resistance profile	Resistance category
AML, AMP, NA, SXT, TE	1	Multidrug resistant
AML, AMP, SXT, TE, F	1	Multidrug resistant
AML, AMP, TE, F	1	Multidrug resistant
AML, KF, NA, F	1	Multidrug resistant
AML, AMP, KF	2	Drug resistant
AMP, TE, F	2	Multidrug resistant
AML, AMP	1	Drug resistant
AML, KF	1	Drug resistant
AML, F	1	Drug resistant
KF	1	Drug resistant
TE	1	Drug resistant

**Key**: AML = Amoxacillin, AMP = Ampicillin, F = Nitrofurantoin, KF = Cephalothin, CRO = Ceftriaxone, SXT = Sulphamethoxazole-trimetoprime, TE = Tetracycline, NA = Nalidixic Acid, CN = Gentamycin.

## Discussion

The current study documented that 3.1% of food handlers had *Salmonella* infection which is consistent with 3.4% reported from Addis Ababa [12] and 2.3% from Ghana [13]. However; this was lower than 7% [14], 13.63% [15], and 18% [16] prevalence from Jakarta (Indonesia), Addis Ababa (Addis Ababa), and Nigeria, respectively. The lower prevalence rate in the present study might be due to the fact that food handlers in University of Gondar had a better habit of hand washing after using a toilet and before handling of food than food handlers participated in other studies aforementioned above.

On the other hand, lower prevalence rates of *Salmonella* infection were reported from different studies compared to this study. In these reports there were no *Salmonella* infection among food handlers working in a student’s cafeteria of University of Gondar and Gondar Teachers Training Collage [17], in food workers of Turkey [18], in food handlers at Hawassa [5], and food workers in a medical college in North India [19]. In another studies, 0.93% and 0.03% *Salmonella* infection rates were recorded from Northern Ethiopia asymptomatic food handlers [20] and amongst food workers in hotels, supermarket, food factories, and restaurants in Japan [21], respectively. The sample size and personal hygiene differences of the food handlers might help to explain this discrepancy.

The high prevalence of *Salmonella* in the age group of 18–37 years in the present study was comparable with a study conducted in Addis Ababa [12]. This might be due to the fact that the chronic *Salmonellae* carrier state occurs most commonly among middle age women [22]. In addition, the prevalence of *Salmonella* infection was higher in females in the current investigation; supported by the previous studies in Ethiopia [12] and Ghana [13].

The results of antimicrobial susceptibility test in the current research revealed that nearly half of (46.2%) the isolates were multidrug-resistant according to the recent drug-resistance definition [23]. In addition, all of the *Salmonella* isolates were resistant for at least one or more drugs tested. Further analysis of antimicrobial susceptibility test results showed that 69.2% of *Salmonella* isolates were resistant to Amoxicillin which conform a report from Nigeria [16] that proved *Salmonella* species in recent years have become progressively more resistant to clinically useful antibiotics including Amoxicillin. The resistant nature of *Salmonella* isolates to Amoxicillin might be ascribed to high level of utilization of this drug due to its relatively cheaper price and readily available nature to the local community in the current study area. This might create the opportunity for misuse of this drug thus heralding the emergence of resistant strains of *Salmonella*. Furthermore, 54.8% *Salmonellae* isolates were resistant to ampicillin. This finding was in agreement with findings from Jimma and Addis Ababa that reported 54%, [6] and 59.4% [7] resistant isolates, in that order. But this ampicillin resistance pattern of *Salmonella* isolated during this study was lower from reports that indicated 100% [15] and 82.3% [24] resistance in Addis Ababa isolated from dairy farm attendant and pediatric patients, respectively. Furthermore; the current result was lower than 87.5%, 100%, and 91.8% of ampicillin resistant *Salmonella* isolates reported from Addis Ababa [12], Bahir Dar [25] and Tamil Nadu [26], respectively.

The 46.2% tetracycline resistant *Salmonella* isolates registered in our research was in agreement with 46.9% [7], but higher than 33.3% [15] resistance reports from Addis Ababa. Yet it is by far lower than 92.9% resistance disclosed from USA [27]. Tetracycline is the most frequently prescribed drug in the current study area including for other infectious diseases both in human and veterinary medicine that could also be mentioned as one of the reasons for the development of such a higher resistance. A worrying aspect of the current result is that it will also be difficult to treat other clinical infectious cases using tetracycline. Although the resistance pattern of *Salmonella* isolates from food handlers for sulphamethoxazole-trimetoprim in this study is low (15.4%), previous studies have documented much higher [24, 7] and lower [15] resistance rates from Ethiopia in different times.

Interestingly, all of the *Salmonella* isolates in the current investigation were susceptible to gentamycin and ceftriaxone, which is in agreement with similar studies in Lagos [28] and Ethiopia [12]. This result was higher than 75% [15] and 19.8% [7] susceptibility reports for gentamycin and 78.8% [7] for cetriaxone from Addis Ababa. ceftriaxone is relatively recently introduced and more costly drug compared to cephalothin in the current study area. Due to this, the community might have used it less frequently that contributed for the low rate of resistance recorded. Good efficacy was also observed in nalidixic acid in which 84.6% of the isolates were susceptible that was nearly in line with the study conducted in Lagos, Nigeria [28] and Tamil Nadu, India [29]. In contrast, a study conducted in Ghana revealed resistance to nalidixic at a higher level [30]. These differences to nalidixic acid efficacy might be due to presence or absence of previous exposure of isolates to the drug in other countries than this study area or it might be due to the fact that nalidixic acid is costly and only rarely prescribed in our study area. On the other hand, 46.2% of the isolates exhibited resistance to nitrofurantoin, which is higher than 15% resistance profile reported from Addis Ababa [15].

## Conclusion

Drug resistant including multidrug-resistant *Salmonella* isolates are circulating among food handlers at the University of Gondar posing high risk of infection for the University community having close contact with those carriers. Programmed medical checkup for food handlers with respect to infectious diseases was not practiced at the University. Health education and check-up, and immediate appropriate treatment strategy for positives based on *in-vitro* antimicrobial susceptibility tests should be in place. Further in-depth serotyping and drug resistant gene identification should be carried out.

## Authors’ information

Authors are human and animal health professionals and members of Zoonosis and Food Safety research team at the University of Gondar. They are engaged and interested in Zoonotic Food-borne Pathogens and Food Safety research.
